# Efficacy, Safety, and Acceptability of Misoprostol in the Treatment of Incomplete Miscarriage: A Systematic Review and Meta-analysis

**DOI:** 10.1055/s-0043-1776029

**Published:** 2023-12-23

**Authors:** Thiago Menezes da Silva, Moema Alves Guerra de Araujo, Ana Carolina Zimmermann Simões, Ronnier de Oliveira, Kleyton Santos de Medeiros, Ayane Cristine Sarmento, Robinson Dias de Medeiros, Ana Paula Ferreira Costa, Ana Katherine Gonçalves

**Affiliations:** 1 Maternidade Escola Januário Cicco, Universidade Federal do Rio Grande do Norte, Natal, RN, Brazil; 2 Centro de Ciências da Saúde, Universidade Federal do Rio Grande do Norte, Natal, RN, Brazil; 3 Instituto de Ensino, Pesquisa e Inovação, Liga Contra o Câncer, Natal, RN, Brazil; 4 Department of Obstetrics and Gynecology, Universidade Federal do Rio Grande do Norte, Natal, RN, Brazil

**Keywords:** Abortion, Miscarriage, Misoprostol, Curettage, Aborto, Aborto espontâneo, Misoprostol, Curetagem

## Abstract

**Objective** To assess the efficacy, safety, and acceptability of misoprostol in the treatment of incomplete miscarriage.

**Data sources** The PubMed, Scopus, Embase, Web of Science, Cochrane Library, and Clinical Trials databases (clinicaltrials.gov) were searched for the relevant articles, and search strategies were developed using a combination of thematic Medical Subject Headings terms and text words. The last search was conducted on July 4, 2022. No language restrictions were applied.

**Selection of studies** Randomized clinical trials with patients of gestational age up to 6/7 weeks with a diagnosis of incomplete abortion and who were managed with at least 1 of the 3 types of treatment studied were included. A total of 8,087 studies were screened.

**Data collection** Data were synthesized using the statistical package Review Manager V.5.1 (The Cochrane Collaboration, Oxford, United Kingdom). For dichotomous outcomes, the odds ratio (OR) and 95% confidence interval (CI) were derived for each study. Heterogeneity between the trial results was evaluated using the standard test, I ^2^ statistic.

**Data synthesis** When comparing misoprostol with medical vacuum aspiration (MVA), the rate of complete abortion was higher in the MVA group (OR = 0.16; 95%CI = 0.07–0.36). Hemorrhage or heavy bleeding was more common in the misoprostol group (OR = 3.00; 95%CI = 1.96–4.59), but pain after treatment was more common in patients treated with MVA (OR = 0.65; 95%CI = 0.52–0.80). No statistically significant differences were observed in the general acceptability of the treatments.

**Conclusion** Misoprostol has been determined as a safe option with good acceptance by patients.

## Resumo

**Objetivo** Avaliar a eficácia, segurança e aceitabilidade do misoprostol no tratamento do aborto incompleto.

**Fontes de dados** Os bancos de dados PubMed, Scopus, Embase, Web of Science, Cochrane Library e bancos de dados de Ensaios Clínicos (clinicaltrials.gov) foram pesquisados para os artigos relevantes, e estratégias de busca foram desenvolvidas usando uma combinação de termos temáticos de Medical Subject Headings e palavras de texto. A última pesquisa foi realizada em 4 de julho de 2022. Nenhuma restrição de idioma foi aplicada.

**Seleção dos estudos** Foram incluídos ensaios clínicos randomizados com pacientes com idade gestacional até 6/7 semanas com diagnóstico de aborto incompleto e que foram manejadas com pelo menos um dos três tipos de tratamento estudados. Um total de 8.087 estudos foram selecionados.

**Coleta de dados** Os dados foram sintetizados usando o pacote estatístico Review Manager V.5.1 (The Cochrane Collaboration, Oxford, United Kingdom). Para resultados dicotômicos, o odds ratio (OR, na sigla em inglês) e o intervalo de confiança (IC) de 95% foram derivados para cada estudo. A heterogeneidade entre os resultados do ensaio foi avaliada usando o teste padrão, estatística I ^2^ .

**Síntese dos dados** Ao comparar misoprostol com aspiração a vácuo médico (MVA, na sigla em inglês), a taxa de aborto completo foi maior no grupo MVA (OR = 0,16; IC95% = 0,07–0,36). Hemorragia ou sangramento intenso foi mais comum no grupo do misoprostol (OR = 3,00; 95%CI = 1,96–4,59), mas a dor após o tratamento foi mais comum em pacientes tratados com MVA (OR = 0,65; 95%CI = 0,52–0,80). Não foram observadas diferenças estatisticamente significativas na aceitabilidade geral dos tratamentos.

**Conclusão** O misoprostol tem se mostrado uma opção segura e com boa aceitação pelos pacientes.

## Introduction

According to estimates from the World Health Organization (WHO), ∼ 55 million abortions occurred between 2010 and 2014 worldwide, with 45% considered unsafe abortions. Africa, Asia, and Latin America account for 97% of the unsafe abortions. 1 The WHO defines unsafe abortion as a procedure for the termination of pregnancy performed by people without the necessary skills. Alternatively, it is defined as a procedure performed in an environment not standardized to perform medical procedures, or a combination of these two factors. Despite scientific advances that allow safe abortions for patients, unsafe abortions continue to occur, causing increased healthcare costs, complications, and maternal deaths. 2 In Brazil, abortion is a public health problem because of its magnitude and persistence. 3

Additionally, several procedures have been performed in the face of abortion, such as pharmacological, surgical, or expectant procedures. 4 Not all forms of treatment are widely available in public services, and not all are accepted by patients. A previous meta-analysis comparing the possible management of first-trimester miscarriages found that one other success was achieved for every three patients treated surgically instead of medically. In contrast, expectant management showed variable efficacy depending on the clinical presentation. 5

The WHO defines abortion as the expulsion or extraction of a conceptus before reaching 22 weeks of gestational age or weighing < 500 grams. 6 Additionally, abortion can be classified and approached in different ways, including gestational age (early or late), clinical presentation (threatened, inevitable, infected, incomplete, complete, or missed), and origin (spontaneous or provoked). 7

Incomplete abortion, the subject of the present study, has an eminent clinical diagnosis and is characterized by transvaginal bleeding associated with an open uterine cervix upon physical examination when the products of conception have not been wholly discharged. 5 This is the most frequent clinical presentation of this condition. 7 8 9

Currently, misoprostol (prostaglandin E2 analog), along with mifepristone, is the reference drug for medicated uterine emptying in cases of spontaneous or induced abortion, both in the first gestational trimester and at more advanced gestational ages. However, mifepristone is unavailable in Brazil. 4 5 Misoprostol works by inducing the uterus to contract and expel the remaining tissues, with no immediate necessity for operating theatres, sterile equipment, or skilled personnel. Thus, it is even more relevant in low-resource settings. Therefore, the present systematic review and meta-analysis aimed to assess the efficacy, safety, and acceptability of misoprostol in the treatment of incomplete miscarriage. Managing these patients using a less invasive option may be essential.

## Methods

The present systematic review was designed and reported according to the Preferred Reporting Items for Systematic Reviews and Meta-Analyses (PRISMA). 10 As this study is based on published studies, no ethical approval or patient consent was required.

The study protocol was registered in the PROSPERO International Prospective Register of Systematic Reviews (registration number: CRD42018116776).

The design of the present study followed the PICOS strategy for systematic reviews as follows: Population (P): patients with incomplete miscarriage diagnosed up to 6/7 weeks of gestation; Intervention (I): treatment with misoprostol; Comparator (C): a manual vacuum aspiration (MVA) or curettage; Outcomes (O): Efficacy: complete evacuation of the uterus, with no need for additional intervention; Safety: adverse effect profile (frequency and severity); Acceptability: patient's overall satisfaction and if she would choose the same method again; Study design (S): randomized controlled trials (RCTs).

The articles were searched in the PubMed/MEDLINE, SCOPUS, EMBASE, Web of Science, Cochrane Library, and Clinical Trials databases using the following combinations of Medical Subject Heading terms and Boolean operators: “( *pregnancy* OR *pregnant* *women* OR *abortion* OR *miscarriage* ) AND ( *misoprostol* OR *dilatation* OR *curettage* OR *vacuum* OR *aspiration* ) AND ( *Randomized controlled trial* OR *blind method* OR *trial* OR *RCT* ).” The final search was conducted on January 15, 2021, and updated on July 4, 2022. No language restrictions were applied. The keyword details and complete search strategy are provided in Supplemental file 1.

The inclusion criteria were randomized clinical trials with patients of gestational age up to 13 weeks and 6 days, diagnosed with incomplete abortion and managed with at least one of the three types of treatment studied. Nonrandomized studies and those that analyzed nonspontaneous abortions or patients not in the first trimester were excluded from the review.

Two researchers, TMS and ACZS, independently screened the studies according to their titles and abstracts. Duplicate studies were excluded, and the full text was reviewed to determine whether they met the selection criteria. A third researcher, ACAS, resolved the disagreements between the reviewers regarding the inclusion of an article. Once the studies were selected, the data of each were summarized in a single spreadsheet to standardize all the results obtained; the latter process was conducted by two researchers, MAGA and RO. Any missing data would have been retrieved by contacting the corresponding author or their coauthors through phone or e-mail, but there was none.

The following data were included in the spreadsheet: author, year of study, country in which the study was conducted, number of patients enrolled, age of patients, gestational age, number of patients assigned to each treatment method, main adverse effects reported, satisfaction with the technique, and follow-up time.

One of the researchers, AKG, used the Cochrane risk-of-bias analysis tool 11 to assess randomization, participant allocation, participant-practitioner blinding, and outcome assessment. Incomplete data and possible conflicts of interests were also considered. The risk of bias was assessed according to the predetermined criteria: low, high, or uncertain.

Data were synthesized by another researcher, APFC, using the statistical package in Review Manager V.5.1. For dichotomous outcomes, the OR and 95%CI were derived for each study. The heterogeneity between the trial results was evaluated using a standard test with *p* = 0.1 and the I ^2^ statistic, which is a quantitative measure of inconsistency across studies, with 0% indicating no observed heterogeneity and values of 50% indicating substantial heterogeneity. When heterogeneity was measured (I ^2^ = 75%), a random-effects model was used to combine the trials and calculate the relative risk (RR) and 95%CI using the DerSimonian and Laird algorithm in a meta-analysis package for R. The other study characteristics and results have been summarized.

The quality of evidence of the studies was evaluated by one of the researchers, KSM, according to the Grading of Recommendations Assessment, Development, and Evaluation (GRADE). 12

## Results

### Study Selection

**Fig. 1:**
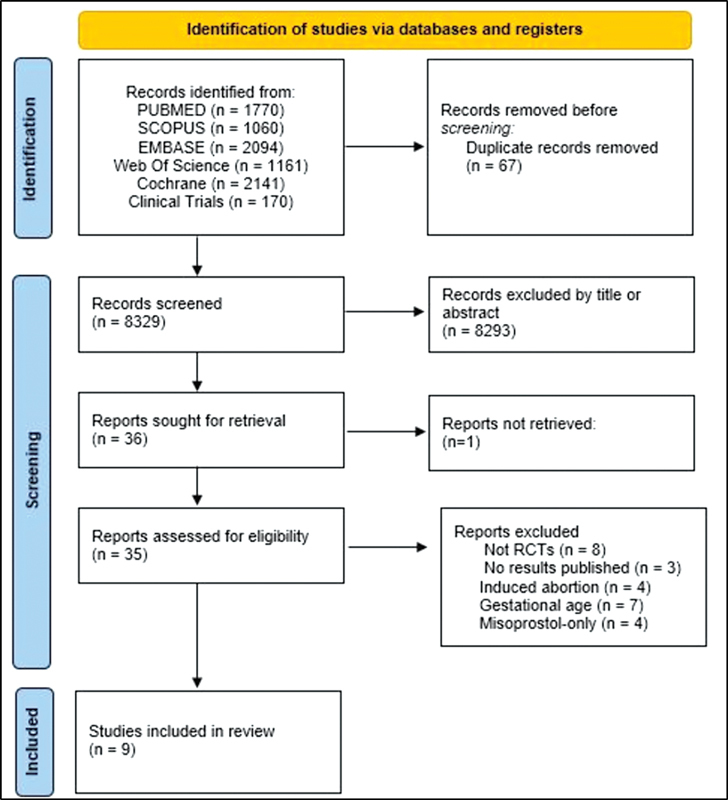
PubMed search strategy.

Database searches identified 8,087 articles ( Fig. 1 ). From this initial amount, 67 were excluded due to duplication, 7,985 were excluded after title and abstract review, 1 study could not be retrieved, and 21 were excluded because they did not meet the eligibility criteria. Case reports and series were excluded from the present study. Ultimately, nine studies met the eligibility criteria and were included in the final review ( Chart 1 ).

**Chart 1:** Characteristics of the studies included in the systematic review

Author, year	Country	Study sesign	Sample	Average age of participants (years old)	Gestational age inclu ded (weeks)	Intervention	Groups of comparison (n° of patients)		Follow-up time (days)	Outcomes
							**Misoprostol**	**Manual vacuum aspiration (MVA)**		
**Dabash R. et al, 2010** 24	Egypt	RCT	695	28	0–12	400mcg sublingual x MVA	348	347	7	Favours MVA in safety.
**R. Montesinos et al., 2011** 22	Ecuador	RCT	203	18-33	0–12	600 mcg oral x MVA	106	97	7	Favours MVA in safety.
**Taylor J. et al, 2011** 26	Ghana	RCT	218	26	0–12	600 mcg oral x MVA	108	110	7–14	Favours misoprostol in acceptability.
**Chigbu B. et al, 2012** 25	Nigeria	RCT	320	29	0–12	600 mcg oral x MVA	160	160	7–14	Favours misoprostol in acceptability.
**Shochet T. et al, 2012** 28	Nigeria, Niger, Senegal, Burkina Faso, Mauritania	RCT	839	28	0–12	400 mcg sublingual x surgical evacuation	465	374 allocated to “surgical evacuation” (Curettage or MVA)	7–14	Favours “surgical evacuation” in efficacy and safety.
**Das CM. et al., 2014** 20	Pakistan	RCT	222	28	0–12	600 mcg x MVA	111	111	7	No difference between groups.
**Ibyiemi KF. et al, 2019** 21	Nigeria	RCT	198	28	0–13	600 mcg oral x MVA	100	98	7	Favours MVA in efficacy and safety (except for “pain”).
**Ani VC. et. al, 2022** 27	Nigeria	RCT	203	29	First trimester (9.1 ± 2.0)	400mcg sublingual x MVA	102	101	7	Favours MVA in efficacy, favours misoprostol in safety (specifically in pain).
**Nwafor et al, 2021** 23	Nigeria	RCT	94	18–45	0–13	600 mcg oral x MVA	48	46	7	Favours misoprostol in acceptability.

Abbreviation: MVA, manual vacuum aspiration.

Throughout the search and selection of studies, seven articles, 13 14 15 16 17 18 19 which compared mifepristone and misoprostol, were analyzed. However, these were discarded because they did not meet the inclusion criteria. The studies included patients with a gestational age of 5 to 12 weeks but excluded incomplete abortion from their analysis. These studies concluded that the treatment with mifepristone and misoprostol was more effective than misoprostol alone for the management of missed miscarriages, which was not the objective of our analysis. Nonetheless, given the relevance of this drug in current studies and its future potential as another accessible treatment option, the meta-analysis in question will be addressed in the “Discussion” section.

### Study Characteristics

Of these studies, eight were RCTs comparing treatment with misoprostol and MVA. One multicentric trial compared misoprostol with “surgical evacuation,” which could be MVA or curettage, according to the usual practice of the service. However, there was no information regarding the number of patients allocated to each procedure. A total of 2,992 patients were enrolled, with a mean age of 28.1 years old and gestational age up to 13 6/7 weeks. The follow-up time ranged from 24 hours to 28 days in the study protocols. Eight studies involving 1,950 patients who underwent treatment with either misoprostol or MVA were included for meta-analysis. 20 21 22 23 24 25 26 27

### Outcomes of Misoprostol

**Fig. 2:**
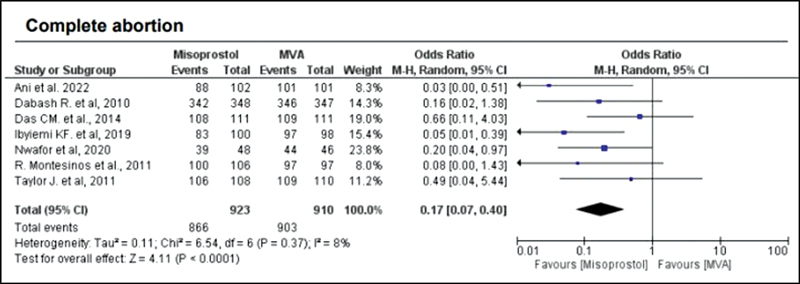
Complete abortion.

**Fig. 3:**
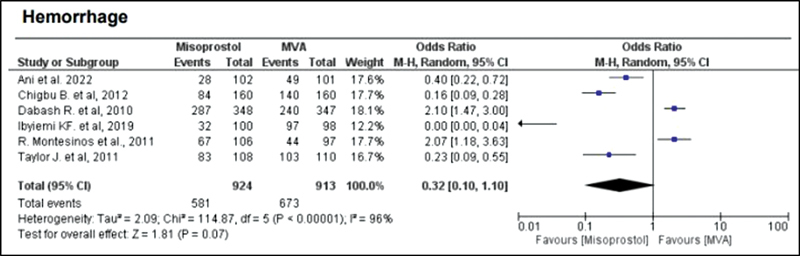
Hemorrhage.

**Fig. 4:**
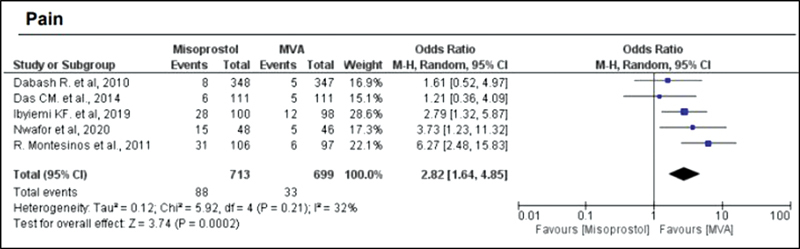
Pain.

**Fig. 5:**
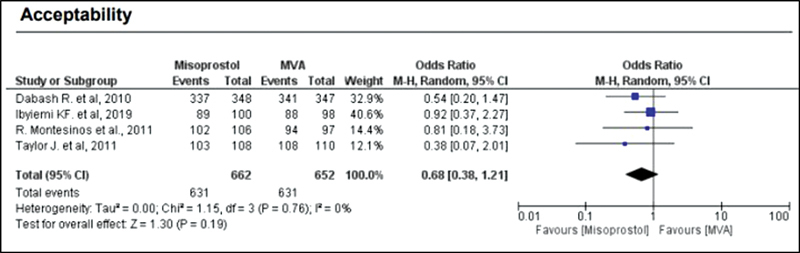
Acceptability.

When comparing misoprostol with MVA, the rate of complete abortion was higher in the MVA group (OR = 0.16; 95%CI = 0.07–0.36) ( Fig. 2 ). Hemorrhage or heavy bleeding was more common in the misoprostol group (OR = 3.00; 95%CI = 1.96–4.59), but pain after treatment was more frequent in patients treated with MVA (OR = 0.65; 95%CI = 0.52–0.80) ( Figs. 3 and 4 ). Regarding the general acceptability of the treatment (in relation to overall satisfaction and if the same method would be chosen again), misoprostol showed an OR of 0.67 with a 95%CI of 0.38–1.19 ( Fig. 5 ).

Studies that could not be included in the meta-analysis were analyzed individually and showed conflicting results. Shochet et al. 28 compared 465 patients who were given 400 μg of sublingual misoprostol with 374 patients undergoing surgical evacuation (MVA or curettage) and observed higher efficacy (risk ratio [RR] = 0.90; CI = 0.88–0.92) and lower rates of hemorrhage (0.6 versus 11.6%) and pain (24.4 versus 54.8%) in the surgical group (p < 0.001). Nonetheless, a higher number of patients in the misoprostol group said that they would choose the same treatment again if needed (97.6 versus 87.8%; *p* < 0.001). No significant difference was noted in overall satisfaction with the method (98.5 versus 98.1% in the misoprostol and surgical groups, respectively; *p* = 0.78).

### Risk of Bias

**Fig. 6:**
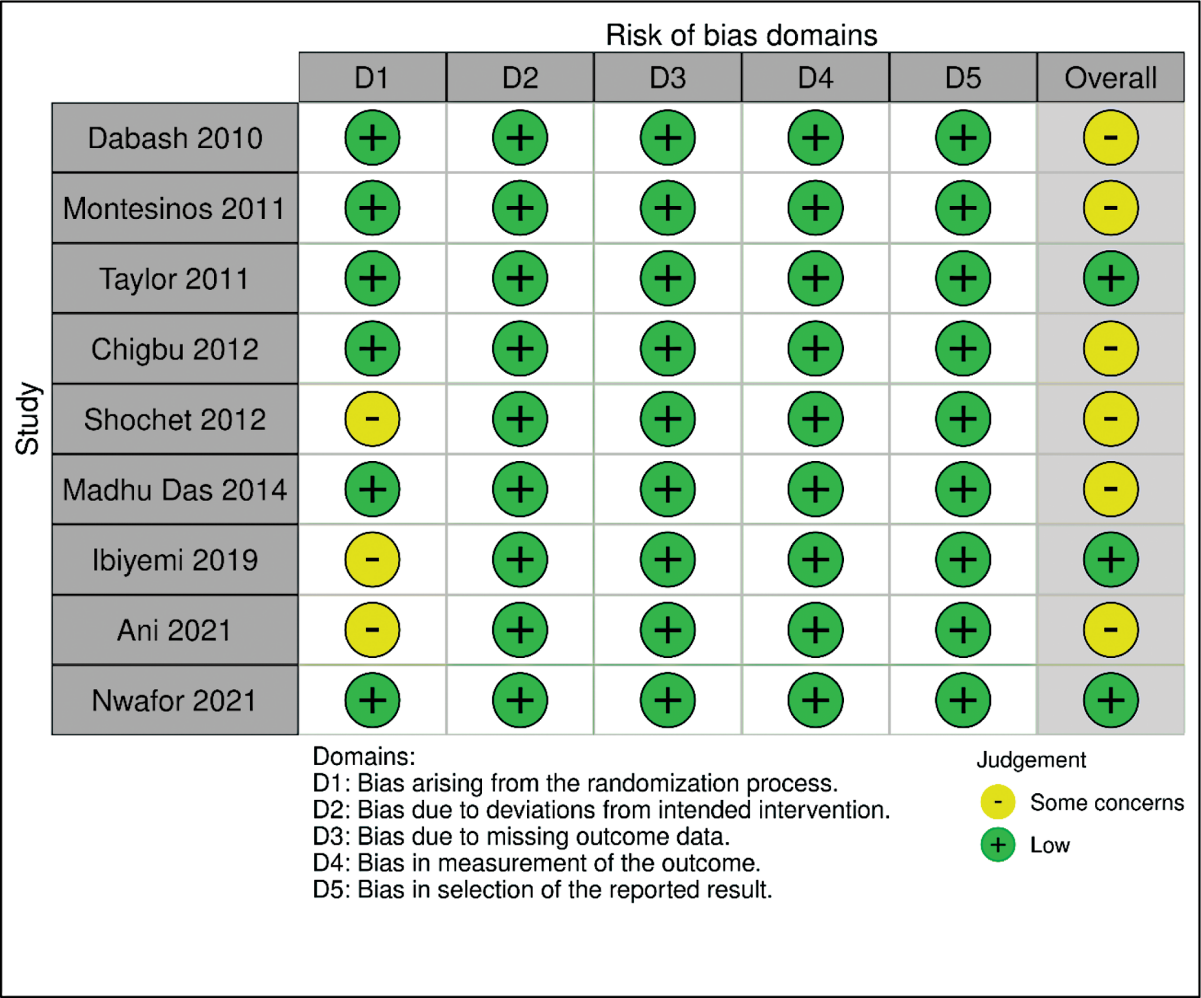
Risk of bias.

In general, the studies presented a low risk of bias. The clinical trials were conducted safely. However, Shochet et al. (2012), Ibiyemi et al. (2019), and Ani et al. (2022) showed some risk of bias in the randomization process ( Fig. 6 ).

### Quality of Evidence

The efficacy and satisfaction of outcomes presented high quality evidence. However, the bleeding/hemorrhage and pain outcomes showed low-quality evidence, mainly due to high heterogeneity between the studies and high CI of the results ( Chart 2 ).

**Chart 2:** GRADE quality of evidence

Certainty assessment							N° of patients		Effect		Certainty	Importance
N° of studies	Study design	Risk of bias	Inconsistency	Indirectness	Imprecision	Other considerations	Misoprostol	Manual vacuum aspiration	Relative (95% CI)	Absolute (95% CI)		
Inducing abortion												
7	randomised trials	not serious	not serious	not serious	not serious	none	923	910	−	**0** (0.07 higher to 0.4 higher)	⨁⨁⨁⨁ High	CRITICAL
Hemorrhage												
5	randomised trials	not serious	serious a	not serious	serious b	none	713	699	−	**0** (1.64 higher to 4.85 higher)	⨁⨁◯◯ Low	CRITICAL
Pain												
6	randomised trials	not serious	very serious c	not serious	not serious	none	924	913	−	**0** (0.1 higher to 1.1 higher)	⨁⨁◯◯ Low	CRITICAL
Acceptability												
4	randomised trials	not serious	not serious	not serious	not serious	none	662	652	−	**0** (0.38 higher to 1.21 higher)	⨁⨁⨁⨁ High	IMPORTANT

Abbreviation: CI: confidence interval.

a. Heterogeneity of 32%; b. 95%Cl: [1.96, 4.59]; c. Heterogeneity of 96%.

## Discussion

Since miscarriage is still a complex health problem, often neglected by health policies, discussion on management options and application of scientific evidence to provide humanized, effective, and safe care that patients will accept is fundamental to alleviate the physical and psychological burden of this event.

In this scenario, misoprostol is a suitable option, although the present meta-analysis revealed that it has a slightly lower efficacy than MVA or curettage, with a higher rate of heavy bleeding. However, the patients reported less pain when using misoprostol and presented similar acceptability as surgical treatments. In addition, misoprostol can be more accessible in low-resource settings, as not all areas have tertiary hospitals with operating rooms where the patients can have proper treatment. Moreover, when treated medically, the patient does not need to be hospitalized for the entire treatment period, as outpatient care seems to be an alternative, 28 which may be better for patients with a good support network at home.

Furthermore, Von Hertzen et al. performed a randomized clinical trial with 2,066 patients who received three doses of misoprostol 800 µg via different administration routes. Their results showed that the commonly reported adverse effects were pain, diarrhea, fever, or chills. In that study, only 0.04% of the patients had vaginal bleeding that required a return to the hospital. 29 Recently, Sheldon et al. also published a randomized clinical trial using only 800 µg of misoprostol for induced abortion. In this study, treatment efficacy ranged from 84 to 87%, and adverse effects (diarrhea, nausea, vomiting, fever, or chills) were self-limiting and well tolerated by patients. Only one woman had vaginal bleeding, which required a return to the hospital for surgical completion. 30

In the usual practice, some professionals are concerned about the occurrence of infection when surgical treatment is not chosen. In 2006, the Miscarriage Treatment (MIST) Trial 31 randomized 1,200 patients diagnosed with a first-trimester miscarriage to be treated surgically, medically, or expectantly. The results showed no difference in the incidence of infection within 14 days of follow-up. A posterior analysis of fertility rates in the three groups showed that medical treatment is safe from a reproductive future point of view. 32 However, advising patients on the alarm signs, such as persistent fever, heavy bleeding, change in mental state, dizziness, and fainting that may appear with an infection, and consulting a health care provider is essential. 33

Concerning the rates of complete evacuation of the uterus after treatment, a network meta-analysis showed that all surgical and clinical methods for managing a miscarriage might be more effective than expectant management and a placebo. Surgical techniques were ranked highest for managing a miscarriage, followed by the clinical approach, which ranked above expectant management and a placebo. Suction aspiration after cervical preparation was the highest-ranking surgical procedure. Expectant management and placebo had the highest chance of serious complications, including the need for unplanned or emergency surgery. A subgroup analysis showed that surgical and clinical methods might be more beneficial in patients with missed miscarriages than in those with incomplete miscarriages. 34

Concerning the limitations of the present study, we can cite: lack of standardization for some of the outcomes considered. For instance, some studies reported “pain” as the number of patients who presented with symptoms after treatment, whereas some reported, using a mean visual analog scale (VAS). In addition, bleeding was sometimes reported according to its intensity and sometimes referred to as any amount of bleeding. Regarding misoprostol, each study had a specific protocol for dosing and route of administration, which may be pointed out as another problem when comparing outcomes in different studies.

To minimize these effects, several strategies were used to test the evidence, such as the assessment of quality and risk of bias.

Once the efficacy, safety, and acceptability of misoprostol in incomplete abortion are well established, future researchers may be interested in finding the ideal route of administration (oral, sublingual, or vaginal), perfect dosage, and intervals of administration when necessary.

## Conclusion

Misoprostol has been determined as a safe option with good patient acceptance. This acceptance may be related to the fact that the patient did not need to be hospitalized and reported less pain. Furthermore, misoprostol appears to be more accessible in low-resource settings. However, the quality of the body of evidence for bleeding/hemorrhage and pain outcomes was “low,” mainly because of the high heterogeneity between the studies and an increased CI effect. Therefore, the results regarding the efficacy of misoprostol in this meta-analysis cannot be generalized.
